# Applications of Computer-Assisted and Robotic Navigation in Anterior Cervical Spine Surgery: A Scoping Review

**DOI:** 10.7759/cureus.114329

**Published:** 2026-08-11

**Authors:** Armando F Pérez Castell, Jose I Fernández-Faudoa, Alfredo Porras Mendoza, Carlos A Arellanes-Chavez

**Affiliations:** 1 Neurosurgery/ Spine Surgery, Star Medica Hospital, CISNE Spine Academy, Chihuahua, MEX; 2 Neurosurgery, Star Medica Hospital, CISNE Spine Academy, Chihuahua, MEX

**Keywords:** anterior cervical spine surgery, computer-assisted navigation, image-guided spine surgery, neuronavigation, robotic navigation

## Abstract

Computer-assisted navigation and robotic guidance have become increasingly adopted in spine surgery to improve anatomical localization, trajectory planning, and implant placement accuracy. However, their application in anterior cervical spine surgery remains limited and heterogeneous. This scoping review aimed to map the available clinical evidence regarding the applications, technological evolution, safety, and reported accuracy of computer-assisted and robotic navigation in anterior cervical procedures. The review was conducted in accordance with the PRISMA extension for Scoping Reviews and the methodological framework proposed by Arksey and O'Malley. PubMed/MEDLINE was searched for English-language clinical studies published between January 2010 and December 2025. Reference lists of included articles were also manually screened. Eligible studies reported the use of computer-assisted navigation or robotic guidance in anterior cervical spine surgery. Study characteristics, surgical indications, navigation platforms, technical strategies, and clinical outcomes were extracted and qualitatively synthesized. Fourteen studies met the eligibility criteria. The evidence consisted predominantly of case reports and small case series, with only a limited number of retrospective observational studies. Navigation was used for anterior cervical discectomy and fusion, corpectomy, transcorporeal microforaminotomy, odontoid screw fixation, and complex anterior instrumentation. O-arm-based navigation and StealthStation were the most frequently reported platforms. Alternative reference-frame strategies included Mayfield-, Caspar retractor-, and clavicle-mounted systems. The included studies generally reported technically successful procedures, acceptable implant positioning, neurological or symptomatic improvement, and few complications; however, outcome definitions and follow-up were inconsistent. Computer-assisted and robotic navigation appear feasible for selected anterior cervical procedures, particularly in anatomically complex cases. Nevertheless, the available evidence remains limited, heterogeneous, and predominantly low-level. Prospective comparative studies using standardized outcome measures are required before definitive conclusions regarding clinical superiority, long-term effectiveness, or cost-effectiveness can be established.

## Introduction and background

Computer-assisted navigation (CAN) has become one of the most important technological advances in modern spine surgery, improving surgical precision, implant placement, and patient safety. Since its introduction in the mid-1990s, navigation has evolved from fluoroscopy-based systems to sophisticated three-dimensional (3D) image-guided platforms capable of providing real-time anatomical visualization and instrument tracking during surgery [1-4].

The incorporation of intraoperative computed tomography (CT), O-arm-based intraoperative imaging, and robotic-assisted systems has further expanded its applications, particularly in complex anatomical regions and minimally invasive procedures [1,4-6]. Although computer-assisted navigation and robotic-assisted surgery share the common objective of improving surgical accuracy, they represent distinct technological approaches. Navigation systems may be classified as passive or active. Passive navigation provides real-time intraoperative image guidance and stereotactic feedback without restricting the surgeon's movements, whereas active navigation guides surgical instruments along predefined trajectories. Most contemporary robotic platforms function as active navigation systems by integrating preoperative planning with guided execution to improve surgical accuracy and precision [2-6].

Navigation technologies have transformed spine surgery beyond pedicle screw placement and are now widely applied in deformity correction, spinal oncology, trauma, revision surgery, and minimally invasive procedures. Numerous studies have demonstrated improved implant accuracy, reduced screw malposition, fewer revision procedures, and decreased radiation exposure for both patients and operating room personnel [1,4,5].

Anterior cervical spine surgery presents unique technical challenges because of the close proximity of the spinal cord, vertebral arteries, esophagus, trachea, and other critical neurovascular structures [7,8]. In addition, the cervical spine is characterized by smaller osseous anatomy, greater segmental mobility, and technical difficulties related to reference frame fixation and image registration, making accurate localization and trajectory planning particularly important [3,9,10]. Consequently, even minor deviations during anterior cervical procedures may result in significant neurovascular complications.

Recent advances in navigation technology have improved the feasibility of image-guided anterior cervical surgery. Safe navigation in the supine position has been demonstrated using multiple reference frame strategies, including head fixation devices, Caspar retractor-mounted arrays, and clavicle-based reference systems [7]. Among contemporary platforms, the O-arm has become one of the most widely adopted technologies by combining intraoperative cone-beam CT with automatic image registration, providing real-time navigation, improved lesion localization, greater implant accuracy, and reduced radiation exposure [1,5,11,12]. More recently, robotic platforms such as TiRobot, Tinavi, and Mazor X have further expanded the capabilities of navigation by integrating three-dimensional surgical planning with robotic guidance.

Despite the increasing adoption of navigation and robotic technologies in spine surgery, their application in anterior cervical procedures remains relatively underrepresented compared with posterior cervical and thoracolumbar surgery [9]. Most available evidence consists of case reports and small case series, resulting in considerable heterogeneity regarding pathology, surgical indications, navigation platforms, technical workflows, reference frame strategies, and reported outcomes.

Given the limited volume and heterogeneous nature of the available clinical evidence, a scoping review represents an appropriate methodology to systematically map the existing literature, summarize current clinical applications and technological developments, and identify gaps requiring further investigation. Accordingly, this scoping review aimed to systematically map the available evidence regarding the clinical applications, safety, reported accuracy, and technological evolution of computer-assisted and robotic navigation in anterior cervical spine surgery while identifying current knowledge gaps and future research priorities.

Research question and Population-Concept-Context (PCC) framework

This scoping review was guided by the following research question: What is the current evidence regarding the clinical applications, safety, accuracy, and technological evolution of computer-assisted and robotic navigation in anterior cervical spine surgery?

The review was structured according to the Population-Concept-Context (PCC) framework recommended for scoping reviews. The population was patients undergoing anterior cervical spine surgery; the concept was computer-assisted navigation and robotic navigation systems; and the context was anterior cervical procedures performed for degenerative, traumatic, neoplastic, infectious, or other cervical pathologies.

## Review

Methods

Study Design

This scoping review was conducted in accordance with the Preferred Reporting Items for Systematic Reviews and Meta-Analyses extension for Scoping Reviews (PRISMA-ScR) guidelines and followed the methodological framework proposed by Arksey and O'Malley [13]. The review process included identification of the research question, identification of relevant studies, study selection, data charting, and synthesis of the available evidence.

Search Strategy

A literature search was performed in PubMed/MEDLINE to identify studies evaluating computer-assisted and robotic navigation in anterior cervical spine surgery. PubMed/MEDLINE was selected as the primary database because this represents a highly specialized and heterogeneous field with a relatively limited body of clinical literature. The search included articles published between January 2010 and December 2025.

To maximize study identification, the reference lists of included articles were manually screened, and supplementary manual searches were conducted to identify potentially relevant studies not retrieved during the primary database search. The PubMed search was performed using combinations of free-text keywords related to the surgical approach and navigation technology. The search strategy included terms such as ("anterior cervical spine surgery" OR "anterior cervical discectomy and fusion" OR ACDF OR "odontoid screw fixation" OR "cervical corpectomy") AND ("computer-assisted navigation" OR "image-guided surgery" OR "robotic navigation" OR "robotic-assisted surgery" OR "O-arm" OR "intraoperative navigation" OR StealthStation). Retrieved titles and abstracts were subsequently screened according to the predefined eligibility criteria.

Study Selection

Titles and abstracts were screened according to predefined inclusion and exclusion criteria. Full-text articles were subsequently retrieved and assessed for eligibility. Articles were excluded during full-text review when they did not meet the predefined eligibility criteria regarding study design or surgical approach.

Eligibility Criteria

Eligible studies included original clinical articles reporting the use of computer-assisted navigation or robotic guidance in anterior cervical spine surgery. Only articles published in English between January 2010 and December 2025 were considered. Randomized controlled trials, prospective and retrospective cohort studies, case series, and case reports were eligible for inclusion. Studies were excluded if they were review articles, editorials, conference abstracts, or technical notes without clinical data. Studies involving posterior cervical procedures, thoracic or lumbar surgery, non-clinical investigations, and articles that did not specifically evaluate computer-assisted or robotic navigation in anterior cervical spine surgery were also excluded. Only studies meeting the predefined eligibility criteria were included in the final qualitative synthesis.

Data Extraction

Data extracted from each study included the author and year of publication, study design, number of patients, surgical approach, cervical level treated, underlying pathology, patient age and sex, navigation platform, clinical outcomes, reported complications, and relevant technical observations.

Methodological Quality Assessment

Methodological quality assessment was performed according to study design. Case reports and case series were evaluated using the Joanna Briggs Institute (JBI) Critical Appraisal Checklists, whereas retrospective cohort studies were assessed using the Newcastle-Ottawa Scale (NOS). Quality assessment was performed to characterize the methodological quality of the available evidence and identify potential sources of bias. In accordance with scoping review methodology, no studies were excluded based on their methodological quality assessment.

Results

Study Selection and Characteristics

**Figure 1:**
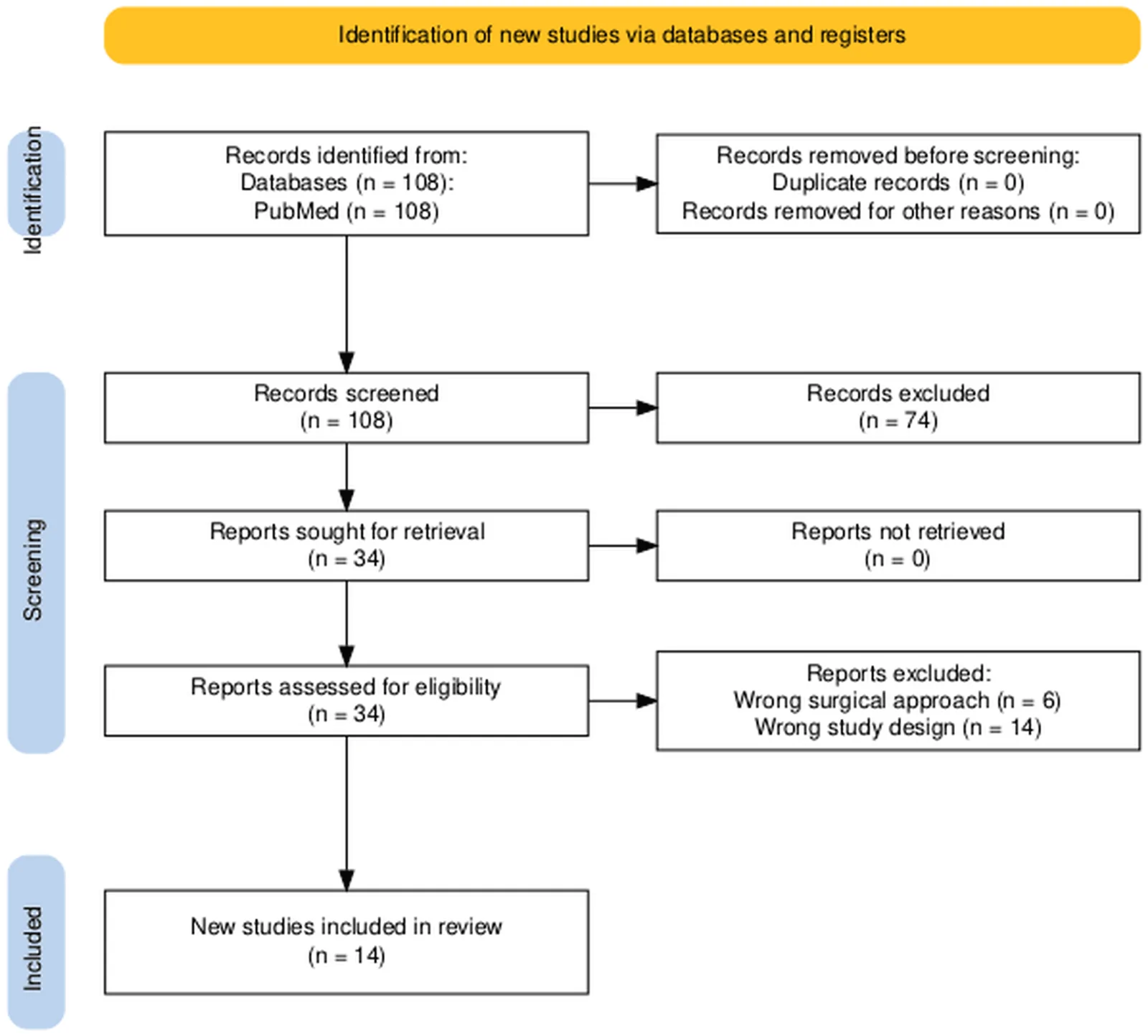
PRISMA-ScR flow diagram of study selection Flow diagram illustrating the identification, screening, eligibility assessment, and inclusion of studies evaluating computer-assisted and robotic navigation in anterior cervical spine surgery. The diagram was generated according to the PRISMA 2020 framework and adapted for scoping reviews. PRISMA-ScR - Preferred Reporting Items for Systematic Reviews and Meta-Analyses extension for Scoping Reviews

A total of 108 records were identified through the database search. After title and abstract screening, 34 articles underwent full-text assessment. Fourteen studies published between 2010 and 2025 met the predefined eligibility criteria and were included in the final qualitative synthesis (Figure 1). The PRISMA-ScR flow diagram summarizes the study selection process, including identification, screening, eligibility assessment, and final inclusion of studies, in accordance with the PRISMA 2020 methodology described by Haddaway et al. (2022) [14]. The available literature consisted predominantly of case reports and case series, with only a limited number of retrospective observational studies, reflecting the highly specialized nature of navigation-assisted anterior cervical spine surgery.

The principal characteristics of the studies, including study design, pathology, surgical approach, navigation platform, and outcomes reported by Kim et al. [14], Lee et al. [15], Castro-Castro et al. [16], Tian et al. [17], Rizk and Ottenbacher [18], Starkweather et al. [19], Asuzu et al. [29], Avrumova et al. [21], Khan et al. [22], Gong et al. [23], Wang et al. [12], and Yamada et al. [24], are summarized in Table 1.

**Table 1:** Characteristics, surgical approaches, navigation systems, and outcomes of the included studies CAN - computer-assisted navigation; ACDF - anterior cervical discectomy and fusion; ACCF - anterior cervical corpectomy and fusion; OPLL - ossification of the posterior longitudinal ligament; MOC - multilevel oblique corpectomy; NR - not reported; M - male; F - female

Author and year	No. cases	Age/Sex	Study Design	Surgical Approach	Level	Pathology	Navigation system	Outcomes	Complications	Navigation benefit (key finding)
Kim J., 2010 [14]	8	NR	Case series	Transcorporeal anterior microforaminotomy	C5-T1	Degenerative	O-arm	Neurological improvement in all patients	Not explicitly reported	Feasible and accurate navigation-assisted transcorporeal microforaminotomy with neurological improvement
Lee H., 2010 [15]	11	NR	Case series	Multilevel oblique Corpectomy (MOC)	C2-C3 C3-T1	Degenerative	StealthStation + C-arm	Faster and more complete MOC	Not explicitly reported	Improved localization and more complete multilevel oblique corpectomy
Castro-Castro J., 2014 [16]	5	80% male (4/5)	Case series	Axial anterior cervical spine	Type II Odontoid fracture (C2)	Fracture	O-arm	100% screw placement accuracy; 80% fusion rate	Not explicitly reported	Accurate odontoid screw placement (100%) with safe trajectory guidance
Pirris S., 2014 [7]	22	NR	Case series	Subaxial anterior cervical spine	C4-T1	Degenerative	O-arm/BrainLab	Accurate localization of surgical levels and safe execution of ACDF and corpectomy procedures.	None (explicitly reported)	Accurate level localization and safe execution of anterior cervical procedures
Tian W., 2016 [17]	1	61F	Case report	Axial anterior cervical spine	Type II Odontoid fracture (C2)	Fracture	TiRobot	Satisfactory reduction and position of screw	None (explicitly reported)	Successful robotic-assisted odontoid screw fixation with satisfactory reduction
Rizk & Ottenbacher, 2019 [18]	5	4M/1F; mean 59±15y	Case series	Anterior cervical instrumentation	C1–C2 and cervicothoracic (C7–T3)	Traumatic fractures, odontoid fracture, tuberculosis, instrumentation failure, pathological fractures (Osteomalacia)	StealthStation	Accurate screw placement and intraoperative 3D verification of instrumentation	Not explicitly reported	Safe anterior cervical navigation using cranial fixation
Starkweather et al., 2020 [19]	1	86 M	Case report	Anterior cervical spine	Type II odontoid fracture (C2)	Fracture	O-arm	Complete recovery	Not explicitly reported	Accurate placement of dual odontoid screws using O-arm navigation
Asuzu et al., 2021 [20]	1	F (young adult)	Case report	Anterior cervical spine	Type II odontoid fracture (C2)	Fracture	Mazor X	Complete recovery at six-month follow-up	Not explicitly reported	Feasible robotic-assisted anterior cervical screw placement
Tanaka et al., 2021 [11]	6	3M/3F; mean 62.8y	Case series	Anterior cervical approach (ACDF n=3, ACCF n=3)	Subaxial cervical spine C4-C6	Degenerative	O-arm	High navigation accuracy, adequate decompression, and JOA recovery rate of 52.4%	Not explicitly reported	Safe anterior cervical navigation using an alternative workflow.
Avrumova et al., 2024 [21]	1	68M	Case report	Anterior cervical spine	C4-T1	Degenerative (DISH)	CAN	Symptomatic improvement	Not explicitly reported	Accurate osteotomy planning and anatomical localization
Khan et al., 2024 [22]	1	28M	Case report	Anterior odontoid screw fixation	Type II odontoid fracture (C2)	Fracture	Brainlab	Accurate screw placement, successful fixation	Not explicitly reported	Novel navigation-assisted odontoid fixation with accurate screw placement
Gong et al., 2024 [23]	1	64M	Case report	Anterior odontoid screw fixation	Type II odontoid fracture (C2)	Fracture	O-arm + StealthStation	Accurate screw placement, fracture healing	Not explicitly reported	Safe and reproducible odontoid screw fixation technique
Wang et al., 2025 [12]	7	NR	Case series	Anterior odontoid screw fixation	Type II odontoid fracture (C2)	Fracture	Tinavi	100% clinically acceptable screw placement, fracture healing in all patients	None (explicitly reported)	Safe and effective robotic anterior odontoid fixation
Yamada et al., 2025 [24]	5	4M/1F; mean 59±15y	Case series	Anterior cervical surgery	Subaxial cervical spine	Degenerative	StealthStation	High navigation accuracy; no complications	None (explicitly reported)	Feasible intraoperative CT navigation with preserved accuracy

Clinical Applications and Surgical Procedures

The included studies encompassed a broad spectrum of anterior cervical procedures performed with computer-assisted or robotic navigation. Reported surgical techniques included transcorporeal anterior microforaminotomy, multilevel oblique corpectomy, anterior cervical discectomy and fusion (ACDF), anterior cervical corpectomy and fusion (ACCF), anterior odontoid screw fixation, and complex anterior cervical instrumentation.

Clinical indications were heterogeneous and included degenerative cervical disorders, type II odontoid fractures, traumatic cervical injuries, cervical deformities, instrumentation failure, tuberculosis, pathological fractures, ossification of the posterior longitudinal ligament (OPLL), cervical disc herniation, cervical spondylotic amyotrophy, and diffuse idiopathic skeletal hyperostosis (DISH). Degenerative pathology and type II odontoid fractures represented the most frequently reported indications for navigation-assisted anterior cervical surgery. These findings indicate that navigation-assisted techniques have been preferentially applied to technically demanding anterior cervical procedures in which accurate anatomical localization and trajectory planning are critical.

Geographic Distribution of Studies

**Figure 2:**
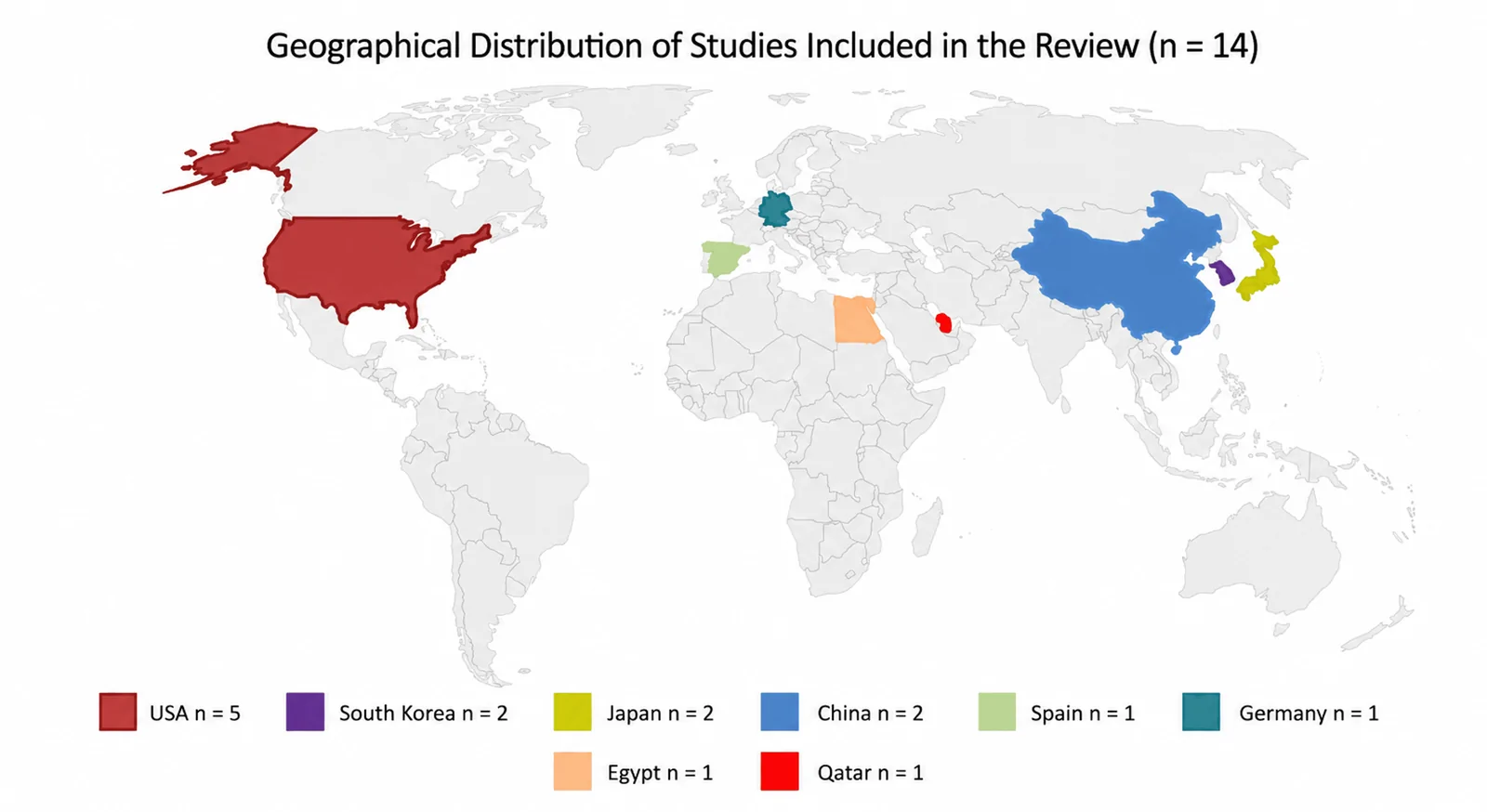
Geographical distribution of studies included in the review World map illustrating the countries in which the 14 included studies were conducted. The United States contributed the highest number of studies (n = 5), followed by China (n = 2), South Korea (n = 2), and Japan (n = 2). Individual studies originated from Spain (n = 1) and Qatar (n = 1), while one study involved collaboration between institutions in Egypt and Germany (n = 1). The figure demonstrates the international distribution of research on computer-assisted and robotic navigation in anterior cervical spine surgery. Figure created by authors using Excel (Microsoft, Redmond, Washington)

The included studies originated from multiple geographic regions, reflecting the growing international interest in navigation-assisted anterior cervical spine surgery. The United States contributed the highest number of studies (n = 5), followed by China (n = 2), South Korea (n = 2), and Japan (n = 2). Individual studies originated from Spain and Qatar, and one study involved collaboration between institutions in Egypt and Germany. This geographical distribution demonstrates the international adoption of computer-assisted and robotic navigation technologies in anterior cervical spine surgery, although the available evidence remains concentrated within a limited number of specialized institutions and high-volume centers. The geographical distribution of the included studies is illustrated in Figure 2. This distribution suggests that published experience with navigation-assisted anterior cervical spine surgery remains concentrated in specialized centers with access to advanced intraoperative imaging and navigation technologies.

Navigation Platforms

**Figure 3:**
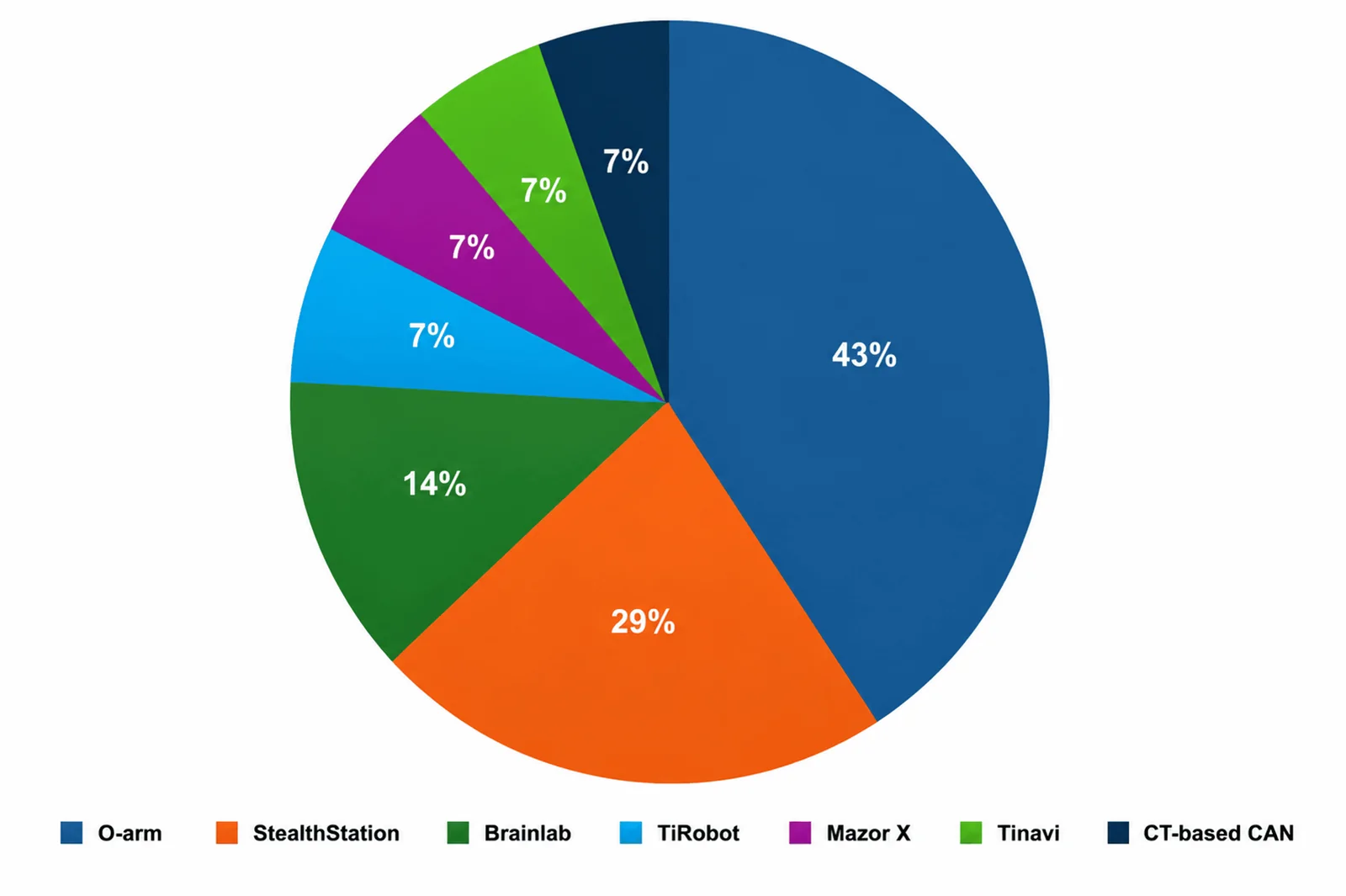
Distribution of navigation platforms among included studies Pie chart showing the relative frequency of navigation technologies reported across the included studies. O-arm and StealthStation were the most commonly utilized platforms.

Multiple navigation technologies were identified across the included studies. The most commonly reported systems were O-arm-based navigation and StealthStation platforms, followed by Brainlab, TiRobot, Tinavi, Mazor X, and other computer-assisted navigation systems. The distribution of navigation platforms identified among the included studies is summarized in Table 2 and graphically illustrated in Figure 3. O-arm-based navigation and StealthStation were the most frequently reported platforms among the included studies, suggesting that intraoperative CT-based navigation remains the predominant technology reported for anterior cervical procedures.

**Table 2:** Distribution of navigation platforms among the included studies CAN - computer-assisted navigation

Navigation platform	Studies
O-arm	Kim et al. [14], Castro-Castro [15], Pirris et al. [7], Starkweather et al. [19], Tanaka et al. [11], Gong et al. [23]
StealthStation	Lee et al. [15], Rizk et al. [18], Gong et al. [23], Yamada et al. [24]
Brainlab	Pirris et al. [7], Khan et al. [23]
TiRobot	Tian et al. [17]
Mazor X	Asuzu et al. [20]
Tinavi	Wang et al. [12]
CT-based CAN system	Avrumova et al. [21]

Several studies also described innovative reference-frame fixation strategies, including cranial fixation through Mayfield head holders, Caspar retractor-mounted arrays, and clavicle-based reference systems designed to improve navigation accuracy during anterior cervical procedures.

Reference-frame fixation strategies varied considerably among the included studies. The most frequently reported method consisted of cranial fixation through a Mayfield head holder, which was utilized in studies by Pirris et al., Rizk and Ottenbacher, Starkweather et al., Khan et al., and Gong et al. [7,18,19,22,23]. Alternative fixation techniques included a Caspar retractor-mounted reference frame described by Tanaka et al. and a novel clavicle-mounted reference frame introduced by Yamada et al. [11,24]. Several robotic navigation systems incorporated proprietary registration methods without external reference-frame fixation. A summary of the reference-frame fixation strategies identified across the included studies is presented in Table 3. The variability in reference-frame fixation strategies reflects the continuous evolution of navigation techniques aimed at maintaining registration accuracy despite the inherent mobility of the cervical spine.

**Table 3:** Reference-frame fixation strategies reported in the included studies Summary of reference-frame locations and registration methods used for computer-assisted and robotic navigation during anterior cervical spine surgery. Most studies employing image-guided navigation used cranial fixation through a Mayfield head holder, whereas alternative strategies included Caspar retractor-mounted and clavicle-mounted reference frames, as well as proprietary robotic registration systems.

Author (year)	Reference frame location
Kim et al. (2010) [14]	Not specified
Lee et al. (2010) [15]	Not specified
Castro-Castro (2014) [16]	Not specified
Pirris et al. (2014) [7]	Mayfield head holder
Tian et al. (2016) [17]	Robotic registration
Rizk & Ottenbacher (2019) [18]	Mayfield head holder
Starkweather et al. (2020) [19]	Mayfield head holder
Asuzu et al. (2021) [20]	Robotic registration
Tanaka et al. (2021) [11]	Caspar retractor
Avrumova et al. (2024) [21]	Not specified
Khan et al. (2024) [22]	Radiolucent Mayfield head holder
Gong et al. (2024) [23]	Mayfield head holder
Wang et al. (2025) [12]	Robotic registration
Yamada et al. (2025) [24]	Clavicle-mounted reference frame

Synthesis of Technology Utilization Patterns

Taken together, the platform and reference-frame data summarized in Tables 1-3 reveal two consistent patterns across the included literature. First, a temporal trend is apparent: earlier studies (2010-2016) relied predominantly on O-arm- and StealthStation-based image-guided navigation paired with Mayfield or Caspar-retractor-mounted reference frames, whereas more recent publications (2021-2025) increasingly incorporated robotic platforms (TiRobot, Tinavi, Mazor X) that rely on proprietary registration rather than external reference-frame fixation. Second, the choice of reference-frame strategy appears closely tied to the navigation platform rather than to the underlying pathology: image-based systems were consistently paired with cranial or retractor-mounted frames, while robotic systems avoided external fixation altogether. Together, these patterns suggest that anterior cervical navigation has progressively shifted from passive, image-guided registration toward active, robot-integrated registration strategies over the study period.

Clinical Outcomes

Overall, individual studies consistently reported favorable clinical outcomes, though these findings should be interpreted as descriptive trends across heterogeneous case reports and series rather than pooled or statistically validated results. Most studies described high navigation accuracy, successful implant or screw placement, adequate decompression, satisfactory fracture healing when applicable, neurological improvement, and symptomatic relief, with a low incidence of intraoperative or postoperative complications.

Across the reviewed literature, navigation-assisted techniques were consistently associated with improved anatomical localization, optimized trajectory planning, and facilitation of technically demanding anterior cervical procedures. Collectively, these findings demonstrate consistent technical feasibility across a broad spectrum of anterior cervical procedures despite the heterogeneity of the included studies.

Heterogeneity of Evidence

Considerable heterogeneity was observed regarding pathology, surgical indication, navigation platform, technical workflow, outcome reporting, and study design. Consequently, quantitative synthesis and meta-analysis were not considered appropriate.

Methodological Quality Assessment

The methodological quality assessment demonstrated that the majority of included studies fulfilled most appraisal domains according to their respective study designs.

Case reports generally provided comprehensive descriptions of patient history, diagnostic evaluation, intervention, and clinical outcomes. Among case series, the most common methodological limitations were the absence of explicit statements regarding consecutive patient inclusion and complete case recruitment. Retrospective cohort studies evaluated using the Newcastle-Ottawa Scale demonstrated moderate-to-high methodological quality. Overall, methodological appraisal demonstrated acceptable reporting quality among the included studies despite the predominance of low-level evidence.

Detailed methodological assessments are presented in Tables 4-6.

**Table 4:** Methodological quality assessment of included case series using the Joanna Briggs Institute (JBI) checklist

Study	Q1	Q2	Q3	Q4	Q5	Q6	Q7	Q8	Q9	Q10
Kim et al., 2010 [14]	Y	Y	Y	U	U	Y	Y	Y	Y	N
Lee et al., 2010 [15]	Y	Y	Y	U	U	Y	Y	Y	Y	Y
Castro-Castro J., 2014 [16]	Y	Y	Y	Y	Y	Y	Y	Y	Y	N
Pirris et al., 2014 [7]	Y	Y	Y	U	U	Y	Y	Y	Y	U
Yamada et al., 2025 [24]	Y	Y	Y	Y	Y	Y	Y	Y	Y	N

**Table 5:** Methodological quality assessment of included case reports using the Joanna Briggs Institute (JBI) checklist

Study	Q1	Q2	Q3	Q4	Q5	Q6	Q7	Q8
Tian et al., 2016 [17]	Y	Y	Y	Y	Y	Y	Y	Y
Starkweather et al., 2020 [19]	Y	Y	Y	Y	Y	Y	Y	Y
Asuzu et al., 2021 [20]	Y	Y	Y	Y	Y	Y	Y	Y
Avrumova et al., 2024 [21]	Y	Y	Y	Y	Y	Y	Y	Y
Khan et al., 2024 [22]	Y	Y	Y	Y	Y	Y	Y	Y
Gong et al., 2024 [23]	Y	Y	Y	Y	Y	Y	Y	Y

**Table 6:** Newcastle-Ottawa Scale (NOS) quality assessment of retrospective cohort studies

Study	Selection (4)	Comparability (2)	Outcome (3)	Total Stars
Rizk et al., 2019 [18]	★★★	★	★★★	★★★★★★★
Tanaka et al., 2021 [11]	★★★★	★	★★★	★★★★★★★★
Wang et al., 2025 [12]	★★★★	★	★★★	★★★★★★★★

Discussion

This scoping review mapped the available literature regarding computer-assisted and robotic navigation in anterior cervical spine surgery and identified a relatively small but steadily growing body of evidence spanning from 2010 to 2025. To our knowledge, this represents the first scoping review specifically focused on computer-assisted and robotic navigation in anterior cervical spine surgery. Although navigation technologies have become widely adopted in thoracolumbar instrumentation, deformity surgery, and minimally invasive procedures, their application in anterior cervical surgery remains comparatively underrepresented. The studies included in this review demonstrate that navigation-assisted anterior cervical procedures are technically feasible and have been successfully applied across a broad spectrum of pathologies, including degenerative disease, odontoid fractures, traumatic instability, cervical deformity, OPLL, instrumentation failure, tuberculosis, and diffuse idiopathic skeletal hyperostosis (DISH). An additional finding of this review is that most published reports originated from single-center experiences, reflecting the early stage of clinical adoption and the limited availability of high-level multicenter evidence.

One of the principal findings of this review is the remarkable evolution of navigation technology over the last decade. Early reports by Kim et al. and Lee et al. demonstrated the feasibility of O-arm and image-guided navigation during transcorporeal microforaminotomy and multilevel oblique corpectomy, respectively, highlighting improved anatomical localization and procedural precision in technically demanding anterior cervical procedures [14,15].

Subsequent technological advances introduced intraoperative CT imaging, automatic registration systems, optical tracking platforms, and robotic guidance, substantially expanding the range of procedures that can be performed with navigational assistance. This progression mirrors the broader evolution of image-guided spine surgery described by Wilson et al., who reported a transition from fluoroscopy-based systems toward sophisticated three-dimensional navigation and robotic platforms capable of real-time instrument tracking and surgical planning [5]. The cervical spine presents unique challenges for navigation because of its small osseous dimensions, segmental mobility, and close proximity to the spinal cord, vertebral arteries, esophagus, and trachea. Even minimal trajectory deviations may result in catastrophic neurovascular injury. For this reason, navigation may provide particular value in cervical procedures compared with other spinal regions. Lange et al. emphasized that computer-assisted navigation improves pedicle screw accuracy, reduces screw malposition, and decreases revision procedures in cervical surgery, recommending the use of intraoperative CT-based registration whenever possible because of the mobility of the cervical spine [9]. Similarly, Tian et al. identified navigation as an important strategy to improve precision, safety, and workflow standardization in spinal surgery [10].

Among the navigation systems identified, O-arm-based platforms were the most frequently reported. O-arm-based navigation was used in several studies included in this review, including those by Kim et al. [14], Castro-Castro et al. [16], Pirris et al. [7], Starkweather et al. [19], Tanaka et al. [11], and Gong et al. [23]. The predominance of O-arm-based navigation among the included studies is likely related to its ability to provide high-quality intraoperative three-dimensional imaging, automatic registration, and immediate verification of implant position. Oertel et al. previously demonstrated that intraoperative three-dimensional imaging substantially improves navigational precision and facilitates accurate screw placement, particularly in anatomically complex situations [1]. Likewise, Pirris et al. reported accurate localization and safe execution of ACDF and corpectomy procedures using O-arm and Brainlab navigation systems in the supine position.

A notable observation from the included literature is the increasing use of innovative reference-frame fixation strategies to overcome the challenges of cervical motion and maintain registration accuracy. Different authors proposed cranial fixation through Mayfield head holders, Caspar retractor-mounted reference arrays, clavicle-mounted systems, and cranial reference frames. Rizk and Ottenbacher utilized a cranial reference arc attached to the Mayfield clamp for complex anterior cervical instrumentation, whereas Tanaka et al. introduced a Caspar retractor-based reference frame during ACDF and ACCF procedures. These technical innovations address one of the most important sources of navigational error in cervical surgery and demonstrate the continuous adaptation of navigation systems to anterior cervical anatomy.

Another important finding is the expanding role of robotic-assisted navigation. Although robotic surgery represented only a minority of included studies, the available reports demonstrated encouraging results. Tian et al. described the use of TiRobot for navigated odontoid screw placement, while Asuzu et al. reported the first robotic-assisted percutaneous fixation of a Hangman fracture using the Mazor-X platform [20].

Robotic systems combine preoperative planning, three-dimensional reconstruction, optical tracking, and guided instrumentation, potentially improving screw accuracy while reducing radiation exposure and operative variability. Recent reviews of robotic spinal surgery have similarly highlighted improved accuracy, shorter learning curves, and reduced radiation exposure, although long-term evidence regarding cost-effectiveness remains limited [2,4].

The favorable outcomes reported across the included studies further support the technical feasibility of navigation-assisted anterior cervical surgery. Most studies described successful implant placement, satisfactory decompression, fracture healing when applicable, neurological improvement, or symptom resolution, with few navigation-related complications. However, these findings should be interpreted cautiously because they derive primarily from descriptive studies with small sample sizes and heterogeneous outcome reporting. For example, Kim et al. reported neurological improvement in all patients undergoing navigated transcorporeal microforaminotomy, while Starkweather et al. demonstrated successful fracture healing following navigated odontoid screw fixation [14,19]. Similarly, Avrumova et al. reported complete resolution of dysphagia following navigation-assisted anterior cervical osteotomy for DISH [21].

Despite these encouraging findings, the current evidence remains limited. Most included publications consisted of case reports and small case series, with only a few retrospective observational studies. Consequently, the overall level of evidence remains low, which is consistent with bibliometric analyses demonstrating that a substantial proportion of navigation literature continues to be composed of level IV and V evidence. The lack of comparative studies, randomized trials, standardized outcome reporting, and long-term follow-up limits the ability to draw definitive conclusions regarding superiority over conventional techniques. Taken together, these findings highlight the current state of the available evidence while identifying important gaps that should be addressed by future comparative investigations.

Future investigations should focus on prospective multicenter studies evaluating navigation-assisted anterior cervical surgery using standardized clinical and radiographic outcome measures. Comparative studies examining conventional fluoroscopy, computer-assisted navigation, and robotic guidance would be particularly valuable. Furthermore, future research should assess cost-effectiveness, learning curves, workflow efficiency, radiation exposure, and long-term patient-reported outcomes. As augmented reality, artificial intelligence, and advanced robotic platforms continue to evolve, these technologies may further expand the role of navigation in anterior cervical surgery [5]. Addressing these gaps will be essential to define the future role of computer-assisted and robotic navigation in anterior cervical spine surgery.

Limitations

This review has several limitations. First, the available literature was limited in both quantity and methodological quality, with most included studies consisting of case reports and small case series. Second, substantial heterogeneity existed regarding pathology, surgical indication, cervical level treated, navigation platform, reference-frame strategy, and outcome reporting, precluding quantitative synthesis or meta-analysis. Third, the literature search was conducted in PubMed/MEDLINE, supplemented by manual screening of the reference lists of included studies. Although PubMed/MEDLINE provides broad coverage of peer-reviewed biomedical and spine surgery literature, studies indexed exclusively in other databases may not have been captured. This may have contributed to the limited number of eligible studies and the geographically concentrated distribution of the available evidence. Nevertheless, this limitation should be interpreted within the context of the objectives of a scoping review, which are to systematically map the available evidence and identify existing knowledge gaps. Fourth, the screening strategy focused exclusively on studies reporting computer-assisted or robotic navigation in anterior cervical spine surgery. Consequently, studies involving posterior cervical procedures, thoracic or lumbar surgery, or navigation systems without a clearly defined anterior cervical application were excluded during title, abstract, and full-text screening. Fifth, several reports lacked standardized outcome measures and long-term follow-up, limiting assessment of durability, reproducibility, and long-term clinical effectiveness. Finally, because navigation and robotic technologies continue to evolve rapidly, some findings may not fully reflect the capabilities of the most recent navigation platforms currently available.

## Conclusions

Computer-assisted and robotic navigation represent promising technologies for anterior cervical spine surgery. The available evidence suggests that navigation-assisted procedures are technically feasible, safe, and capable of improving anatomical localization, trajectory planning, implant placement accuracy, and the execution of complex anterior cervical procedures. O-arm-based systems remain the most frequently reported navigation platform, whereas robotic technologies such as TiRobot and Mazor-X are emerging as valuable alternatives for selected clinical indications. This scoping review demonstrates that the current evidence supports the expanding clinical application of navigation-assisted anterior cervical surgery while highlighting important knowledge gaps related to comparative effectiveness, long-term outcomes, and cost-effectiveness. However, the available literature remains predominantly composed of low-level and heterogeneous evidence. Therefore, well-designed prospective multicenter comparative studies are needed to better define the long-term clinical value, cost-effectiveness, and optimal role of computer-assisted and robotic navigation in anterior cervical spine surgery.
